# (Re)Introducing Vygotsky’s Thought: From Historical Overview to Contemporary Psychology

**DOI:** 10.3389/fpsyg.2019.01515

**Published:** 2019-08-07

**Authors:** Olga Vasileva, Natalia Balyasnikova

**Affiliations:** ^1^Psychology Department, Simon Fraser University, Vancouver, BC, Canada; ^2^Department of Language and Literacy Education, University of British Columbia, Vancouver, BC, Canada

**Keywords:** psychological science, history of psychology, Vygotsky, systems approach, language development, culture, developmental science

## Abstract

Theories formulated by Russian psychologist and educator Lev Vygotsky currently range from being applied and celebrated across multiple contexts to be considered outdated. In this paper, we maintain that such inconsistency in application stems from the overreliance on translated or reformulated Vygotskian theories, the attempts to understand these ideas in isolation from the scientific historical context of their development, and the impact of Vygotsky’s personal life circumstances on the development of his scholarship. It is known that Vygotsky’s untimely death prevented him from elaborating on his theoretical views and expanding his early empirical work. We suggest that Vygotsky’s scholarship could be better understood in light of the core principles that transcend all aspects of his work. In this paper, we elaborate on two such core principles: theories of language development and their relation to the integrated systemic approach to psychological development. We argue that although linguistic and historical boundaries have shaped the common perception of Vygotskian theories in anglophone research in a specific way, there is a potential for a renewed application of these theories to modern psychology that might be especially relevant in light of the increasingly interdisciplinary character of the modern science. To support our argument, we provide a brief overview and examples of potential connections between Vygotsky’s scholarship with contemporary landscape in psychological science. The paper presents a brief introduction to the topic of Vygotskian work and its application to modern psychology, rather than an addition to the field of Vygotskian scholarship. It is geared toward non-Vygotskian scholars and invites researchers working in interdisciplinary areas of psychology.

## Introduction

Concepts developed by Vygotsky have transcended time and geographical boundaries. Today, his work is widely applied to many fields of inquiry ranging from psychology (Saxe, 1990/2015; Burman, 2016) to language education (Lantolf, 1994; Lantolf et al., 2018). While this embrace of the Soviet psychologist’s thought is a cause for celebration, a number of scholars have stressed the lack of application of Vygotskian thought to contemporary psychological research (Stetsenko, 2016). As scholars working in interdisciplinary fields ourselves, we believe in the potential for broader applications of Vygotsky’s work. At the same time, we would like to acknowledge an extensive field of Vygotskian scholarship that exists to this day. Indeed, those specialists who have in-depth knowledge of his work and theory will not likely find anything new or surprising in this paper. Our intent is not to outline the Vygotskian project in its entirety, as his research embraced topics spanning from clinical aspects of development to applied aspects of educational practices; neither do we provide a nuanced application of Vygotsky’s work to specific research questions nor present a detailed review of how Vygotsky’s work has been implemented previously. Rather, we seek to inspire interdisciplinary conversations and encourage non-Vygotskian specialists to engage with his work and consider its relevance for their own research. In fact, we have intentionally situated this article in an open access journal as an act of knowledge brokering between these different disciplines and different scholars. With this intent in mind, we do not limit our discussion to one particular aspect of Vygotsky’s work. We intentionally organized our paper around broadly defined research themes. We hope that this would help the readers to identify those areas of research where they could apply Vygotsky’s work or engage with Vygotsky’s ideas.

To assist our readers, we begin with a brief overview of the context of Vygotsky’s work that had significant influence on the way it can be understood today. We maintain that Vygotsky’s work should be conceptualized holistically, as a research program that was broad in scope and governed by fundamental research questions on the nature and development of the human mind. To this end, we address the two core principles of Vygotsky’s work: the systemic approach to the study of mind and the social origin of the mind. We conclude with some recommendations regarding pertinent applications of Vygotsky’s work as it relates to contemporary research in psychology. The end result is thus a text that is rather eclectic conceptually. We encourage our readers to address different parts of the text that they might find more relevant or interesting and further engage with some in-depth accounts of his work provided by Vygotskian scholars.

## Applications of Vygotsky’s Work: Issues and Challenges

Vygotsky has been dubbed the “Mozart of psychology” (Toulmin, 1978). However, his original work has often being rewritten or modified by other “composers” by adjusting or sampling the original work, especially in cases of translation. Therefore, it might be useful to understand how Vygotsky’s work has been taken up by the global research community and, to continue the metaphor, *how* his music (that is, his theoretical contribution and research questions) was performed and *by whom*. For example, we have observed that translated works of Vygotsky that are widely available today are often a de-contextualized aggregation of texts, which had a complex history of publication in their original Russian. While the historical context of his scholarship is fascinating and warrants a separate discussion beyond the limits of this paper, we feel that a brief historical overview of Vygotskian research is needed to situate this paper within modern psychological discourse.

First, as noted by Van der Veer and Yasnitsky (2016a,b) and Zavershneva (2016), a complete and accurate bibliography of Vygotsky’s work is yet to be created. The existing Vygotsky’s bibliographies often contain significant limitations (Van der Veer and Yasnitsky, 2016a,b). As the result, we cannot accurately establish how much Vygotsky wrote himself. It is known that Vygotsky’s texts were not always written and sometimes consisted of notes rather than well-formulated and polished texts, and a significant portion of what is known as Vygotsky’s work was published posthumously. In other words, Vygotsky did not collect and organize these specific pieces of text in the order in which they are found today. Moreover, as noted by Yasnitsky (2011a), some texts that appear under Vygotsky’s name were redacted prior to publication while in other texts portions from different manuscripts were inserted. Furthermore, a significant portion of Vygotsky’s work has not been published at all, and these archival works have become accessible to a broader audience only recently (Zavershneva, 2010, 2014, 2016).

Second, Vygotsky’s texts underwent various levels of censorship during his life and posthumously. It is a well-known fact that in the Soviet Union, research papers were severely censored to make them more agreeable to Marxist ideology. In fact, Vygotsky’s own students censored some of his texts when they rose to prominence in the field during the 1960s and 1970s (e.g., Luria, Leontiev). Consequently, these scholars used Vygotsky’s texts to promote their own work, omitting the parts that were less relevant to their respective research paradigms and in so doing contributed to the establishment of so-called canon of “Vygotsky school” (Fraser and Yasnitsky, 2016; Yasnitsky, 2016a).

Third, publications of Vygotsky’s work in the Western press resulted in significant changes to the original work due to language editing. For example, Cole, John-Steiner, Scribner and Souberman, the editors of *Mind in Society* (Cole et al., 1978) acknowledged that they took “significant liberties” with the original texts while preparing them for publication. This appears to have been done partly to make them more “digestible” and understandable for a Western audience.

The notion of translation is extremely important for the appropriate presentation of Vygotsky’s ideas to a non-Russian-speaking audience, and careful translations of Vygotsky’s texts are currently becoming an area of study in its own right. We refer interested readers to the work of Van der Veer and Yasnitsky (2016a,b) for a review of core Vygotskian semantics. Readers can also refer to Zavershneva (2014) for a “phraseological toolkit” that can assist a reader with essential terminology of Vygotskian texts. More significantly, in our opinion, the translation of Vygotsky’s original Russian texts into other languages adds a layer of difficulty to the accurate application of his work. We maintain that Vygotskian texts could be better understood if studied within the context of the Russian school of psychology and the specific terminology and apparatus of this school. For example, differences in the semantics of the core concepts “mind,” “psyche,” and “cognition” in Russian and English open the door for interpretation, as was highlighted by Bruner (1962), who used the word “*image*” for the Russian word “*znak.*” We feel that the usage of “*image*” in this context could lead readers to different connotations that are rather distant from the more appropriate word “*sign.*”

In sum, when Vygotsky’s texts were adapted by his students, translated, and published in international journals and books, some pieces of his “music material” were omitted. Other pieces “sang” too loudly. However, this situation is not the only obstacle to interpreting Vygotsky for Western readers. What is crucially important is that Vygotsky was writing his “music” during a particular time. This period is important both in the larger historical context and the specific context of developments in the field of psychology, which occurred differently in the West and Soviet Union. As a result, some notes and musical pieces might be unclear to listeners unaware of the specific contexts of their creation.

## The Contexts of Vygotsky’s Work

Researchers have defined at least three major periods of Vygotsky work. The early period took place at the start of the 1920s. The middle period began in the mid-1920s and continued until late 1927 or early 1928 at which time Vygotsky significantly reevaluated his earlier ideas. The final and arguably the most productive period began in 1929 and continued until mid-1934. By this point, Vygotsky’s health was rapidly deteriorating, and he was well aware that he might not live long enough to finish his work. This period for Vygotsky was characterized by a more focused theoretical work and less concern for the meticulous testing of newly developed hypotheses. We suggest that the seemingly lack of precise experimental work is not attributed to any perceived unimportance on Vygotsky’s part. Indeed, this focus on theoretical work in the last period of his life may be better explained by his desire to leave enough theoretical instruments and ideas to his students, who could in turn develop and test his ideas experimentally.

Regarding the specifics of Vygotsky’s life as a researcher, we suggest that a reader of *early* and *late* Vygotsky will not necessarily encounter the *same* scholar. The three major periods of Vygotsky’s work demonstrate varied levels of focus on different topics, changing research programs, and shifting theoretical perspectives. Consequently, we suggest that a single, unified, well-developed theory of Vygotskian theory and methodology is untenable. Rather, there are “several Vygotskies” (Van der Veer and Yasnitsky, 2016a,b), depending on the period of Vygotsky’s work. Moreover, as Toulmin (1978) notes, Vygotsky’s work in psychology was unfinished; he did not have an opportunity to refine his ideas and present them in any final, exhaustive theoretical form. However, our interpretation of Vygotsky suggests that while his career took different turns over the years, it was always guided by general, fundamental questions, such as the structure and origins of human mind. We maintain that other aspects of Vygotsky’s work, such as his interest in defectology, abnormal psychology, and experimental work in comparative psychology, should be understood as leading to an answer for his ultimate research puzzle: the genesis of human mind.

In our opinion, a more accurate understanding of Vygotsky is impossible without an appreciation of the temporal context, which framed Vygotsky’s work and the global social contexts of psychological sciences at that time^1^. We begin our discussion with the focus on *temporal contexts.*

### Temporal Contexts

Vygotsky started his research career soon after the October Revolution of 1917, which impacted the entire trajectory of his career. The revolution brought tremendous changes to every aspect of Russian social life, including research and academic endeavors. The revolution impacted science and academia in Soviet society in at least two major ways: the newly established government threw their support behind scientific research, including research on child development, and established a political ideology that impacted all aspects of Russian society, including science. Since the government established the rules, the new ideology defined what a “right” or “correct” society was expected to look like, how it was supposed to function, and what individuals in the society were supposed to do. In the academy, limits were imposed on what scientists could claim in their research, how phenomena could be examined and explained, and what conclusions were in line with the established ideological framework^2^.

Guided by the ideology of the time, Vygotsky embarked on a metaphorical boat that was sailing toward a new goal – the creation of a new man in a new socialist society. Large populations, such as factory workers, women, and agricultural workers, who previously had limited access to schooling, were now able to pursue an education. Tasked with educating such a large number of people who were illiterate or minimally literate, the Soviet educational system grew exponentially. This increase in the number of individuals seeking an education required innovative scientific approaches, both regarding general science of child development and applied approaches. The old ways of being had been abolished and the new society required different approaches to education, namely a shift in the science of child development. Within this context, Vygotsky’s work was marked by a significant paradigm shift, both in the system of social class interaction and by fundamental shifts in scientific inquiry.

Throughout his entire career, Vygotsky’s work was influenced and constrained by the changing political climate. For example, in 1929, he embarked on a research expedition to Soviet Central Asia together with Luria and a group of students. Despite coming to interesting results, upon completion of the research, Vygotsky was unable to publish and share his work. Even Luria himself, as a well-respected scholar in the Soviet academy, could not publish their joint conclusions until many years later (Lamdan and Yasnitsky, 2016). A possible cause for this stagnation was that the interpretation of the data was not likely favorable for the young Soviet government and did not meet the guidelines set by the ideology of the political changes in the region. Another example of the contextual limitations on Vygotsky’s work was the fact that it could not be published due to the naming of specific individuals in his manuscripts. The 1930s and the decade that followed were characterized by increasing tendencies of isolation from Western science. This led to the demise of extensive reliance on foreign research programs, methods, and theories by Soviet scholars. At the time, extensive citation of foreign names in the work of Soviet scholars was perceived as unpatriotic and in certain contexts could in fact be detrimental to one’s career. Readers are likely familiar with the name Leon Trotsky, a once celebrated communist who was subsequently proclaimed an enemy of the regime. Vygotsky cited Trotsky in some of his manuscripts, and it is apparently this citation that barred the work from publication (Fraser and Yasnitsky, 2016). It is necessary to note, however, that although this situation impacted Vygotsky’s work, it cannot be perceived as an absolute. Vygotsky himself (and his students after his death) collaborated with foreign scholars. Thus, their research never developed in complete isolation from Western psychology (Yasnitsky, 2012a,b, 2016b).

Further examples of contextual influences can be seen in the trajectory of Vygotsky’s research on child development. As mentioned above, the Soviet educational system was tasked with educating large groups of underprivileged children (war orphans, poor or working-class children, and the homeless). All these children had to become new members of the society: they had to be educated, socialized, and provided with professional training that could make them into productive members of the society. As such, the Bolshevik government supported scientific research, discovery and understanding of the basic laws of development, and application of this knowledge for the development of practical methods of education and rehabilitation for underprivileged children. This led to development of a new research discipline – pedology. The science of pedology – a particular direction in Soviet research, which aimed to unite the approaches of various sciences (medicine, biology, psychology, etc.) to the method of child development – was tasked with meeting this challenge. Pedology provided a scientific foundation for the entire educational system, aiming to discover and understand the basic laws of development. Pedology sought to apply this knowledge to practical methods of education and rehabilitation for underprivileged children. The mandate was to make these children “normal” members of society despite their initial impoverished backgrounds. The belief was that should these children be provided with the right social environment, such as an educational system attuned to their needs, the hardships the children have experienced might not have profound, unchangeable consequences for their future.

Overall, the relationship between Vygotsky’s work and political climate of the time was a tumultuous one. The primacy of the environment over innate tendencies was dear to the Bolshevik government. Thus, Vygotsky’s work, with its focus on the social origins of the mind and the environmental impact upon it, was initially well received. With governmental support, pedological institutions were established throughout the country and new classes for teachers and educators were opened. Vygotsky himself considered pedology of the utmost importance in his own work, as the discipline dealt with questions that were central to his interests. However, despite such a favorable start, the discipline of pedology failed to thrive in the Soviet Union (Kozulin, 1984). It became an outcast, and the government halted pedological research.

Regardless of the government’s reasoning, the decline of pedology had a profound impact on Vygotsky’s work. Researchers with a vested interest in pedology had to disassociate themselves from the discipline. Some shifted their research programs to defectology (a discipline more closely aligned with special education and investigating development of children with visual, hearing, and mental impairments); some focused on child clinical psychology and psychiatry; and others turned to physiology or pedagogical research (Yaroshevsky, 1993). This massive departure from pedology did not occur rapidly; however, it had a profound impact on the careers of many researchers. Previous research programs were not halted entirely but had to be significantly transformed. Scholars were required to reformulate their research questions and methodology and corroborate with new host disciplines. Much more significant was the fragmentation of previously established research groups. Members now found themselves in different institutions affiliated with different disciplines. As a result of these changes, the close-knit Vygotskian Circle formed in 1927–1931 eventually dispersed throughout different disciplines and geographical locations (Yasnitsky, 2016b).

Therefore, we conclude that the context of time had a deep impact on the development of Vygotsky’s school of thought. By decontextualizing Vygotskian theories, contemporary researchers might overlook the ideology which guided his work. Moreover, by selectively applying Vygotsky’s ideas to the contexts of their work, many researchers may risk overlooking the changes that Vygotsky made to his own theories.

### Social Contexts

The nature of Vygotsky’s collaboration with colleagues and students had a profound impact on his science. This is why we argue that one needs to acknowledge the social context of Vygotsky’s work as foundational to the development of his theories.

First, it is known that Vygotsky worked closely with a number of like-minded researchers – both his peers and students – and conducted joint experiments with them. The core of the so-called Vygotskian Circle consisted of the *B*ig *Three* – Vygotsky himself, Luria, and Leontiev – and the *Big Five* – first generation students of the three: Zaporozhets, Bozhovich, Levina, Morozova, and Slavina. All of them spent years working intensely with Vygotsky and were clearly influenced by his ideas^3^. The cross-pollination of ideas sometimes gets omitted in viewing Vygotsky as an autonomous, independent thinker.

Second, it is important to consider the trajectory of Vygotsky’s work as it was taken up after his death. After Vygotsky’s passing in 1934, his colleagues continued working on their own research programs, bringing Vygotskian thinking to wider audiences. Vygotsky’s ideas and fundamental beliefs did not truly vanish with the author’s death but continued to develop in later years. The Vygotskian Circle was instrumental in publishing and promoting Vygotsky work posthumously (especially Luria, who contributed to translating Vygotsky’s work in English and disseminating those ideas to the international research community). Several of Vygotsky’s students published their own works shortly after his death (see for example Sakharov, 1994). Although these students published content under their own names, the works were based on close collaboration with Vygotsky or research he conducted himself. Consequently, we can consider some of these publications to be Vygotsky’s work to a large extent. By focusing exclusively on works authored by Vygotsky alone, we could be excluding a large corpus of research that is *de facto* an extension of his original thought.

All Vygotskian Circle researchers have developed their own research programs, as we have mentioned earlier. For example, Luria further worked in clinical psychology and psychopathology, studying memory and aphasia, and Leontiev developed his Activity Theory (Leontiev, 1978). Thus, not all aspects of the Vygotskian Circle are a direct continuation of Vygotsky’s ideas. Some features of their theories are less dependent on Vygotsky and represent a departure from his way of thinking. In some cases, this separation started even before Vygotsky’s death and was known to Vygotsky himself. For example, scholars working with the Vygotsky archive (Zavershneva, 2010, 2016) have found notes, stating that he perceived Leontiev’s development of Activity Theory as a departure from Vygotsky’s own ideas. We would like to reiterate that the Vygotskian Circle can and should be viewed as a development of Vygotsky’s thinking and not simply as its linear extension. Indeed, within the works of the members of the Circle, there are multiple varied engagements with Vygotsky’s original thought. While some of Vygotsky’s colleagues stayed close to his original ideas, others took a significant departure from them.

## Core Principles of Vygotsky’s Work

In section 3 of the paper, we discussed various reasons preventing Vygotsky from finalizing his theory in the complete operational form. We suggest, however, that despite changes and shifting focuses in research, Vygotsky has always followed two core principles: systemic approach to the development of mind and the role of language in this process. In our opinion, these principles are crucial for understanding Vygotsky’s overall theoretical standing and the motivation for his work. Thus, we suggest that those who are embarking on the application of Vygotskian work in their own practice might benefit primarily from understanding and operationalizing these core principles, instead of addressing specific aspects of Vygotsky’s work or rely on his published texts. Moreover, we suggest that incorporation of these two principles has profound implications for about every aspect of psychology. To this end, we discuss how these principles can be applied in various branches of modern psychology as well as other disciplines, such as education and language studies.

### The First Core Principle: Systemic Approach to the Development of the Mind

The first core principle of Vygotsky’s approach to the research of psychological functioning is the application of a systems perspective. Vygotsky continuously stressed that it is a mistake to study psychological functions individually, e.g., to study development of memory or development of perception. He argued that researchers should address the change in the relationships between various functions (Luria and Vygotsky, 1930/1992). In other words, according to Vygotsky, the new object of research should be not *individual functions*, but *psychological systems*.

A systemic approach in the sciences generally stands in opposition to the reductionist approach (Toomela, 2015). In the field of psychology, for example, the reductionist approach is commonly associated with Cartesian thinking and modular approaches (Toomela, 2003). While a discussion of this opposition is beyond the scope of this paper, it is worth noting that Cartesian thinking generally postulates a separation of studies of mind and body, which in modern scientific discourse means an opposition between psychological and biological processes. What is important to consider, given the focus of this paper, is that Vygotsky clearly did not embrace this dichotomy. For him, a developing organism (for example, a human being) could not be reduced to either its biological or social environment. A comprehensive study of child development for Vygotsky had to employ a systemic approach that included both bio-social and bio-psychological aspects of development.

Drawing on a systemic approach in sciences, Vygotsky viewed a unified system as consisting of interdependent elements (Toomela, 2015). In this system, every element has properties that are subject to that system’s function, and no element can be changed without affecting the whole system. At the same time, the system itself is viewed as more than a collection of its individual elements, which change properties when they become parts of a system. Following the systemic principle, Vygotsky saw elements of the human mind as having precursors in animal cognition (Zorina and Smirnova, 2006). However, he argued that the human mind becomes *qualitatively* different once it becomes semiotically mediated. That is, the basic processes of psychological functioning (e.g., memory, decision-making, formation of behavioral programs) acquire new properties as they are mediated through symbols. Emotions, affect, decision-making, and memory acquire specific qualities once they become a part of the general cognitive system. An important aspect of a system is its hierarchical organization. This means that elements comprising a system are interdependent and organized in a particular manner, with some elements being subordinate to other either structurally or functionally. In the earlier period of his work, Vygotsky believed that psychological functions in the human mind are also organized in a hierarchical system. He distinguished between elementary, primitive, lower psychological functions (LPF) that humans share with other animals (e.g., mammals), such as memory and concept formation, and higher psychological functions (HPF) that are characterized by semiotically mediated processes and include decision-making, speech and language, and cultural transmission of knowledge. Vygotsky suggested that in the course of individual development, initially isolated LPF merge with developmentally older HPF.

Some criticism of Vygotsky’s work might stem from ignoring or misinterpreting the systemic principle. For example, Wertsch and Tulviste (1992) take issue with Vygotsky for not explaining how LPF affect HPF in development. As Toomela (2014a) points out, such criticism in fact ignores the systemic approach taken by Vygotsky and applies Cartesian cause-effect thinking. In Vygotsky’s interpretation, mental functions comprising the system change qualitatively in development. This is why within this framework it is impossible to state with certainty how LPF affect HPF, as the former cease to exist in the previous form and become different, culturally mediated psychological processes.

The application of a systemic approach lead Vygotsky to another very important conclusion: since psychological functions are organized in hierarchical systems, developmental processes become *central* for understanding the human mind. The crucial role of developmental processes in the system as a way to understand the system itself is a direct consequence of a principle of systemic organization: when a component becomes part of a system, both the properties of the new whole and the properties of the component change (Vygotsky, 1932/1960; Koffka, 1935; Kohler, 1947). Vygotsky argued that once new components enter the system, they affect the system in general and all other components of this system accordingly. For example, once a child masters language, its psychological functions become semiotically mediated and thus change their qualities, becoming higher psychological functions. This principle was essential for Vygotsky, who maintained that the structure of the mind cannot be understood by researching the mind of an adult. To know what a mind system is, we need to observe mind development in a child. It is not enough to observe only the final product of these processes. The system structure and functionality can only be understood *through system development*. In the following section, we highlight how Vygotsky’s systemic approach to understanding of developmental processes has been utilized across disciplines.

#### Applications of the First Principle

As mentioned above, Vygotsky stressed the importance of understanding developmental processes as a system. Indeed, this is relevant to several psychological disciplines and interdisciplinary research today.

The first area where systemic principle is applicable is *developmental research*. In contemporary developmental psychology, Vygotsky’s systemic perspective comes in contrast with nativism, which suggests that pre-linguistic infants possess a number of psychological abilities in the early stages of development (Onishi and Baillargeon, 2005; Hamlin, 2013, etc.). Nativist studies are unified by a core-knowledge theory framework, postulating that human psychological abilities are largely innate or pre-formed. Nativists postulate that psychological processes in individual development increase quantitatively rather than change qualitatively. According to Vygotsky’s systemic perspective, psychological processes are organized as hierarchical and interconnected dynamic systems, which provide for the heterogeneous nature of the human mind (Vygotsky, 1931/1983a,b; Vygotsky and Luria, 1931/1994). As such, he suggested that not all psychological mechanisms emerge simultaneously but that one’s existing psychological abilities develop qualitatively. Since the nativist perspective suggests that psychological processes change quantitatively, from a systemic perspective, it is a-developmental. If psychological abilities are present from birth and development is conceptualized as quantitative addition of computational power, nativist perspective has difficulties explaining observed variations in development. As Subbotsky (2014) puts it, it is not clear, why a 6-year-old does not demonstrate certain reasoning abilities, when an infant (according to the nativist perspective) does. Similarly, a number of scholars (e.g., Carpendale et al., 2013) expressed criticism to the nativist approach, suggesting that it lacks an explanation of truly developmental processes.

Taking up Vygotsky’s theories, memory development in a 2-year-old, a 3-year-old, and a 5-year-old cannot simply be measured by “how much” memory is “added” at each developmental stage. In the systemic framework, the structure of memory for a 5-year-old is *qualitatively* different from the memory of a 3-year-old. Therefore, instead of studying the development of memory, as a uniform ability throughout childhood, in Vygotskian framework researchers can address formation of “qualitatively different memories.” A systemic framework also calls into question a common approach adopted by nativism research methodology, namely, the search for the initial stages of a given psychological ability in development. Questions such as at what age morality appears or whether preverbal infants understand false-beliefs – adopted by this framework – are meaningless in a systemic perspective. The “morality” of an 8-month-old infant is structurally and functionally different from the morality of a 5-year-old, and both would differ from that of an adult. In a systemic approach, rather than questioning the age at which ability X emerges, it is more appropriate to investigate which aspects of ability X change as they enter other functional systems, to what extend do they change, and under what environmental influences.

The problem of qualitative change in development discussed by Vygotsky relates to a hotly debated question in psychology: whether or not the discipline has methodological tools that can adequately capture developmental processes. Vygotsky (1997) noted that in an attempt to analyze the mind, psychological science tends to dissect its higher forms and structures into primary elements, ignoring the problem of quality, which cannot be reduced to quantitative differences. We believe modern psychology would benefit from applying the Vygotskian standard to research. Valsiner and Van der Veer (2014) suggest that the application of Vygotsky’s Zone of Proximal Development (ZPD) at the conceptual level might provide developmental psychologists and educators with a new approach to assessing and measuring developmental processes. Valsiner and Van der Veer’s suggestions echo the current discussion of the replication crisis in psychology (Earp and Trafimow, 2015) and a call for revisioning methods and statistical apparatus widely used in psychology today. Some researchers suggest this crisis might be resolved with improvements in general theory and the adoption of alternative statistical techniques. For example, Valsiner and Van der Veer (2014) and Toomela (2015) argue that psychology has adopted inferential statistics that are conceptually inadequate for investigating psychological processes. Similarly, a number of developmental researchers (van de Schoot et al., 2014) argue for the adoption of Bayesian statistics in developmental research.

At least two major discussions in Vygotsky’s *neuroscientific approach* are the structural composition of the brain and the relationship between the brain and its environment. Advancements in modern neuropsychology and neuroscience are indeed remarkable; however, some researchers criticize modern neuropsychology for its reductionist approach to human cognition (Toomela, 2014b). Specifically, this criticism focuses on two main aspects of reductionism: the structural organization of psychological processes in the brain and an implied assumption that a careful description of the brain’s structure can be enough for our understanding of human psychology. We suggest that Vygotsky’s scholarship can be useful in addressing such criticism. As Toomela (2014b) suggests, in the search for a neurological basis of behavior, researchers tend to focus on determining which area of the brain is responsible for processing psychological function X. This approach is associated with a modular account of the human brain structure and the assumption that it is possible to determine (more or less precisely) a relationship between specific brain areas and respective psychological processes. Theories on the “modular mind” vary from the more extreme (e.g., “the Swiss-army knife”) model of the mind (Pinker, 1997) to more inclusive (Gazzaniga, 2004; Geary, 2005). However, the underlying assumptions tend to remain the same. Research programs aim to localize precise areas of the cortex responsible for processing specific information or genes that are expressed in a given brain region and in turn affect formation of a particular behavioral program. Such genocentric and modular approaches to the human mind clearly collide with Vygotsky and Luria’s thinking. They suggest that HPF – the most developed, complex, and semiotically mediated psychological processes – are not strictly localized in the brain but are rather dynamically distributed. This approach does not suggest that no localized processes exist in a brain whatsoever. However, similar to modern evidence (Anderson, 2014), it suggests that many complex psychological processes are neither functionally nor structurally localized in the brain. Vygotsky was critical of Pavlov, who associated cognition with the prefrontal cortex and the frontal lobes of the brain. Vygotsky argued that while frontal lobes are important for cognitive processes, some aspects of these processes could be more precisely localized by activating a given area in the brain. According to Vygotsky, real-time processes activate the whole brain and the body, as they activate various functional systems. In this paradigm, complex cognition is not an entity that can be located “somewhere” but rather a process taking part at different locations. For Vygotsky, an appropriate question about cognition is not “where” it can be located but rather “how” it is processed. Vygotsky (1997) wrote that “…no specific function is ever connected with the activity of one single brain center. It is always the product of the integral activity of strictly differentiated, hierarchically interconnected centers.” (p.140). In other words, psychological processes are better explained not by “where” in the brain they are localized, but how different functional brain networks interact with each other in real time. A similar perspective was taken by a number of researchers, including Goldberg (1995), who focused on gradiental approach to neocortical organization and Anokhin (1971), who studied the theory of functional systems, investigating connections between the formation of functional systems and learning. Indeed, modern day neurological research supports Vygotsky’s arguments and shows that many areas of the brain process various types of information (Anderson, 2014). Moreover, studies show that specific areas of the brain – associative cortices – seem to specialize in information synthesis, it is a-modal representation and processing.

Vygotsky (1982) understood the process of development from the neurological perspective, in which the brain reorganizes neuronal connections and creates new, functional systems. In this framework, psychological structures on a neurological level become *functional relations between neurons*, whereas complex functional systems “do not mature by themselves but are formed in the process of communication and material activity of a child” (Luria, 1969, p. 34). This is how the brain, developing as a biological organ, also becomes a cultural organ (Toomela, 2014b). Child interaction with language, cultural tools, artifacts, and social environment leads to changes in the brain, developing new functional systems. Not surprisingly, a prolonged ontogenetic period that in humans is necessary for learning cultural knowledge from conspecifics coincides on a neurological level with a series of massive rewiring events in the brain (de Graaf-Peters and Hadders-Algra, 2006).

The hierarchical nature of functional systems allows for a fresh perspective on understanding impairments in brain damage and developmental disorders. If we conceptualize the damage as impairment in a functional system instead of a broken module, it is possible to separate primary and secondary defects that might affect different levels of the system. Damage to a hierarchically superior level will affect abilities at a lower level as well. Similarly, damage to a lower level of the system will have some effect on that system’s higher levels. For example, Vygotsky demonstrated that impairments in visual perception are related to impairments in language development, verbal thinking, and, in turn, visual thinking (Vygotsky, 1995). What matters is that compensatory mechanisms developed by the brain in each case might differ, depending on the age at which damage to the system occurred (hence the stage of functional system formation). Symptoms that are similar on the surface level might be produced by different psychological processes (e.g., in case of brain lateralization and phonological processing; Stiles et al., 1998). As Akhutina and Shereshevsky (2014) suggest, some modern theoretical perspectives on disabilities, such as neuroconstructivism (Johnson and Karmiloff-Smith, 2004), are frequently in agreement with aspects of Vygotsky and Luria’s work. Drawing on the work of Jean Piaget and systems theorists, neuroconstructivism aims to reconceptualize how we understand development and change from the perspective of a brain. In such reconceptualization, neuroconstructivism applies the systemic thinking so prominent in Vygotsky and Luria’s work with regard to both typical and abnormal development.

Third, systemic approach can benefit *cultural research*. Throughout the 20th century, psychological research focused heavily on the search for “universals” in the human mind. These could be universal rules of learning and stimuli response in behaviorism; the genetic foundation of behavior; innate computational processes in cognitive science and innate modules in evolutionary psychology; and brain structures and processes in neuroscience and neuropsychology. However, recently, cross-cultural researchers have questioned this quest for “universal human nature.” For example, in their seminal paper, Henrich et al. (2010) demonstrate that the majority of samples in psychology research papers come from so-called WEIRD populations. The acronym stands for populations coming from Western Educated Industrialized Rich and Democratic societies. In other words, close to 90% of papers published in psychology are based on investigations of about 12% of the global population. Similarly, Nielsen et al. (2017) have analyzed publications in major developmental psychology journals (Child Development, Developmental Science, and Developmental Psychology), concluding that the majority of all samples in published studies come from North America and Western Europe and are based on English-speaking participants. This situation is troubling. A closer look at “human universals” demonstrates that psychological abilities vary between cultures: executive function (Benson and Sabbagh, 2010), mirror self-recognition (Broesch et al., 2011), infant-directed speech (Broesch and Bryant, 2018), and population-level differences in developmental trajectories by children (Henrich et al., 2010), to name a few.

Vygotskian cultural-historical psychology provides a route for understanding how culture can form mental structures in a more mechanistic way. Specifically, how something *shared* and *social* can become *individual* and *private*. Remembering that Vygotsky defines culture and cultural tools in a particular way is crucial to understanding this point. To illustrate the specificity of his approach, we focus on two perspectives that define culture: cultural-historical psychology based on Vygotskian theory and the socio-cultural approach that is in-line with contemporary Anglo-Saxon and neo-Vygotskian thinking (Matusov, 2008). The socio-cultural approach commonly defines culture as a human-created environment, artifacts, and practices. Toomela (2014b) calls such an approach a-cognitive and a-developmental, as it does not explain how mind structures change qualitatively in development with the use of a cultural tool. Sociocultural schools assign major influence to the formation of behavior to environment. Consequently, since from the perspective of sociocultural school environment determines behavior, there is no need to study individual cognitive levels, as such levels are not informative for the understanding of individual differences in psychological processes. In this framework, development can be explained solely by describing differences in cultural practices. However, as Toomela (2014a,b) discusses, this approach ignores the fact that the same practices are interpreted uniquely by different individuals. This suggests that we cannot understand the source of individual differences solely by relying on the description of cultural practices and avoiding any analysis on an individual cognitive level. In the systemic perspective, an analysis that neglects the individual cognitive level becomes a-developmental. This happens because non-systemic approach does not analyze the individual cognitive development and implies that during development, the mind does not necessarily change qualitatively. Rather, the complexity with which a child interacts with its environment increases. What we wish to highlight is not that cultural psychologists deny that the human mind changes in development, but that the discipline as a whole lacks a mechanistic explanation of how this change happens through interaction with cultural tools. Researchers tend to focus on describing increasingly complex patterns of behavior for a given activity, e.g., social cognition, without necessarily linking these changes to other domains of the mind.

In discussing Vygotsky’s paradigm of cross-cultural research, it is important to mention that Vygotsky’s work was criticized as “ethnocentric” (Matusov, 2008). Vygotsky and Luria’s expeditions to Central Asia, and later Luria’s research with urban, rural, and homeless children (Luria, 1930/1978), aimed to demonstrate developmental changes in cognition introduced by formal education. This work suggested that people indeed tend to apply different problem-solving strategies to a situation depending on whether or not they had formal education (however, see Van der Veer and Yasnitsky (2016a,b) for a critical analysis of these expeditions). Researchers were able to replicate the results of many of these findings later (e.g., Tulviste and Hall, 1991; Subbotsky and Quinteros, 2002). Additionally, modern research acknowledges that the presence or absence of formal education is associated with variability in various psychological phenomena (Saxe, 1990/2015). As Matusov (2008) suggests, the socio-cultural approach opposes Vygotsky’s interpretation of cross-cultural differences. In this framework, humans in different cultures utilize essentially the same psychological mechanisms.

We believe that such criticism of Vygotsky is fair, but only if we do not apply the systemic principle to this problem. In a hierarchically organized mind, some psychological processes are developmentally early, and some emerge later in life. Psychological processes that are associated with the implementation of population-wide formal education, such as reliance on abstract “scientific” concepts of abstract categorization, are developmentally older. In most cultures and for most individuals, all these psychological processes are available, but utilized to varying degrees. In culture A, people may largely rely on *developmentally* early strategies to solve a specific task, while in culture B the same task is commonly solved by means of a *developmentally older mechanism*. The fact that the mechanism utilized in culture A is developmentally younger than the mechanism utilized in culture B does not make it in any way inferior. In such a framework, ethnocentrism can be avoided, as long as we acknowledge (1) the diversity of psychological mechanisms and (2) the absence of the superiority of one mechanism over another based on its developmental emergence. In some cases, developmentally early mechanisms might be more useful for individuals living in a given environment. For example, research demonstrated that in comparison to people living in the West, people with limited formal education in non-Western societies tend to be more accurate at perceptual constancy. Such results might be explained by increased demands to interpret complex spatial environment for the latter group (Ardila and Keating, 2007).

We believe that Vygotsky’s view on culture and its role in the formation of the human mind might generate some interest from within the field of *cultural evolution*, which focuses on an evolutionary understanding of social change. Cultural evolution is a young discipline and is still subject to debates that include the notions of progress in cultural development, cultural relativism, etc. Cultural evolution aims to understand the relationship between culture, environment, and human mind. Importantly, the field attempts to unify behavior, culture, and biology on a conceptual level by studying human development more holistically. It suggests that cultural rather than biological changes have significant effect on both human evolution and the individual development of the human mind. Although the discipline does not rely on Vygotsky’s work explicitly, it raises similar concerns such as the extent to which the human mind and human behavior evolve from cultural processes. We believe researchers working in the field of cultural evolution could find Vygotsky’s view on culture and its role in the formation of the human mind relevant for their work.

### The Second Core Principle: Language and the Social Origins of the Mind

The second core principle of Vygotsky’s work is the social origin of the human semiotically mediated mind and the role of language in this formation. Language is one of the central topics of Vygotsky’s research, and his writings on language development have received much attention on a global scale (Lantolf and Aljaafreh, 1995; Frawley, 1997; Goodman and Goodman, 2014). In this paper, we aim to highlight the connection between Vygotsky’s view of language development and his systemic approach to the development of the mind. It is important to reiterate that specific words and terms coined by Vygotsky (e.g., “sign as a tool”) are not mere metaphors but carry profound meaning and can be better understood in the context of Vygotsky’s general theoretical views. While he saw language as a universal feature of the human species, Vygotsky was mostly interested in the role it played in human development at both the evolutionary and individual levels.

Vygotsky viewed language as an essential component of the human experience, a part of human interaction with the environment, of human behavior and mind and argued that the appearance of language was a driving factor in human development (Vygotsky, 1929/1956). He stressed that through language human psychological processes became semiotically mediated and thus human cognition is fundamentally different from animal one. He also addressed differences in cognition of children before and after they begin to talk. Vygotsky saw a linguistic sign (a word) as a cultural tool closely related to the behavior of a developing organism. He argued that words were a tool similar to physical tools used by children in joint activity with others, as they advance in their development. Importantly, the processes of activity are *first* mastered with an adult and *later* become internalized at the mental level. As such, psychological functions are initially *interpsychic* as an activity between a child and an adult or a more knowledgeable peer and only later become *intrapsychic* as individual thinking of a child. Consequently, Vygotsky devoted much attention to the concept of “inner speech” as a special type of psychological activity and suggests that speech develops first in the social environment and later becomes internalized into mental processes (Friedrich, 2014).

In addition, Vygotsky argued that all linguistic signs are *dual* in nature. On the one hand, their meaning is internalized on the individual level – they become part of the internal psychological processes (Vygotsky, 1931/1983a; Vygotsky, 1960). On the other hand, signs are used in an external activity for social communication and in engagement with others. Therefore, Vygotsky stressed that tools (including linguistic signs) are produced and culturally conventionalized (Arievitch and Stetsenko, 2014). If we accept that meaning of a particular sign is socially conventional then we can conclude that humans can “share minds” by relying on *internal* psychological processes with *socially* constructed meanings. Moreover, human cognition develops on the basis of extracerebral connections. In Vygotsky’s framework, these connections form the basis of regularities created in the *socio-cultural semiotic environment* (Vygotsky, 1982). We believe it is important to understand the difference between this specific conceptualization of the role of language in the development of the mind and a more general notion that humans learn from their social environment.

It was important for Vygotsky to study his research subjects without isolating them from their cultural-developmental context (Toulmin, 1978). Seeing a child’s development as a complex process that happens in a close interaction with his social medium, Vygotsky introduced a new, comprehensive approach that allowed him to observe the child’s psychological and linguistic development during interactions with others (Cole and Gajdamaschko, 2010; Karimi-Aghdam, 2016). Originally, Vygotsky believed that thought and speech performed different functions and evolved relatively independently. He mentions a defined pre-speech phase in the development of intelligence and pre-intellectual phase in the development of speech. He saw similarities in how groups of young children or higher animals communicate without speech (symbols and signs): expressive movements, gestures, facial expressions, etc. However, he emphasized that ways of thinking not associated with speech exist. Vygotsky believed that the age of two is a critical and crucial point in a child’s development. Since at that stage, thought and speech begin to intertwine. Vygotsky observed that this stage is characterized by the rapid increase in the communicative vocabulary of the child. A child first discovers the symbolic function of language, understands the meaning of generalization as a means of communication, and begins to use it for communication and problem solving. As Toomela (2014b) stresses, such an understanding of sign interiorization brings new potential to different research. The concept of “inner speech” as a form of linguistically associated psychological process proposed by Vygotsky has found strong empirical support and is an active field of modern research (Alderson-Day and Fernyhough, 2015; for an excellent review see Sawyer, 2016).

The conceptualization of human environment as first and foremost a social and cultural one is a hallmark of Vygotsky’s work (Stetsenko and Arievitch, 2010; Arievitch and Stetsenko, 2014) and manifests itself in the work of modern developmental researchers (Tomasello, 1999; Rochat, 2001). Tomasello’s work in particular is a prime example of researchers’ focus on social processes and their role in development. Tomasello argues that human-specific psychological abilities develop in human infants due largely to the specificity of a cooperative social environment (Moll and Tomasello, 2007). Quite tellingly, he calls this hypothesis the “Vygotskian Intelligence Hypothesis.” The Vygotskian thesis of the importance of a social environment for the formation of the human mind is concordant with modern research of shared activities, joint attention, development of theory of the mind, development of these abilities through co-joint actions, embodied experiences, and shared routines. Carpendale and Lewis (2004), Racine and Carpendale (2007), and Nelson (2007) argue from a constructivist perspective that the social environment, including a communicative one, actively shapes the mind. Shared routines that are structured, embodied, and rich in meaning provide a foundation for creating shared meaning, knowledge, and feelings. A child’s mind originates in such shared routines that are culturally and linguistically mediated and collaborative (e.g., for an elaborate account of early lexicon development through shared routines, see Nelson, 2003). Such an approach suggests that a child experiences the environment as cultural and semantic from the very onset of life. As Arievitch and Stetsenko (2014) point out, such a view of development is still unorthodox in modern psychology. However, it is a viable approach, if conceptualized through constructivist relations and is in line with Vygotskian thinking.

#### Applications of the Second Principle

As Vygotsky articulated the importance of societal context for education and possible implications for his theory in education, the second principle has been recognized in various disciplines, namely *educational research*. Vygotsky argued that learning is a step ahead, enduring the further development. While completing a challenging task with the help of a teacher or peers, a child develops and learns. Vygotsky’s thinking has a clear application to educational research, for it highlights the agency of various learners and the educational value of a child’s interaction with the environment. The role of the teacher, therefore, is to sustain this interaction and not simply relay decontextualized knowledge. We believe this is a promising expansion of current work in the psychology of learning and classroom research (Moll, 1992, 2013; John-Steiner and Mahn, 2003; Tudge and Scrimsher, 2003; Wells and Claxton, 2008). However, there is a wider application of Vygotsky’s ideas in education far beyond developmental psychology and the theory of childhood speech (Davydov, 1995; Prior and Welling, 2001).

Since Vygotsky was interested in how social medium shapes the cultural values of an individual, his theory is being applied in *intercultural language studies*. Human cultural and linguistic development, Vygotsky writes, passes two stages – first social and then psychological or individual. This transition inwards has its structure and function, as language and culture become internal and fossilized. The most important takeaway from Vygotsky’s theoretical framework for educational research, we believe, is that language structures and speech patterns are internalized through a child’s development. In addition, from this perspective, culture is seen as an aggregate of both material and spiritual values manifested in human behavior. As such, application of Vygotsky’s ideas to the analysis of miscommunication in language teaching allows researchers to postulate that the main cause of misunderstanding does not lie solely in language proficiency. Communication is in itself an expression of one’s lived experience, represented in a symbolic system. While interlocutors might share the code, their use of this code could vary significantly. The underlying source of miscommunication is often the difference in styles of speech and patterns of behavior fossilized in communicants, which interlocutors might be unaware of. Vygotsky called attention to the fact that communication between two individuals is in itself a *communication of consciousness* that occurs in the mind of a carrier of a particular culture and seeks to comprehend the symbols of a different culture.

Moreover, Vygotsky’s focus on the role of the societal context is relevant for modern *developmental science.* Vygotsky proposes two lines of development in a child – a natural line and a cultural one. Such a distinction reflects a conceptual question that transcends psychological science throughout time: the extent to which biology and environment contribute to the development of the mind. Nativism and mainstream cognitivism have been criticized (sometimes explicitly) for separating these lines of development. Followers of alternative perspectives such as constructivism and Developmental Systems Theory (e.g., Oyama, 2000; Gottlieb, 2007; Nelson, 2007; Griffiths et al., 2012) attempt to demonstrate, both theoretically and empirically, that environment cannot be strictly separated from the internal biology of an organism. Rather, the human mind is constructed by active involvement in the environment in which it develops. Gottlieb (2007) describes development as processes interacting at different levels, from cellular to social. Development in this case becomes probabilistic, and any observed patterns can be explained by similarities in developmental contexts rather than hardwired, genetically determined programs. Variations in an environment and its relationship to an individual organism affect the formation of mind structures. In such a framework, there is no one-on-one relation between an organism and environment. Similarly, Vygotsky (1931/1983b) and Vygotsky (1933/1934) argued that environment should be studied not in absolute terms as is, but in relation to a child. The same environment can be different for a 3-year-old and a 5-year-old, as they perceive it differently on an individual level and through different psychological mechanisms. A strong emphasis on environment as a factor in formation of the mind in modern day research is utilized in the ecological approach to cognition (Heft, 2001) and embodied cognition (Shapiro, 2010) and is in line with Vygotsky’s thinking (Karpov, 2005; Falikman, 2014). We believe Vygotskian approach can be applied to DST framework, and an excellent example of such application was developed by Stetsenko (2009).

Furthermore, *modern neuroscience* is often associated with the question of whether an understanding of human brain is sufficient to fully understand the human mind. The answer to this question for many contemporary researchers (e.g., Carpendale et al., 2010; Karmiloff-Smith, 2018) (and we speculate Vygotsky as well) is “no.” As Toomela (2014b) suggests, reductionist approach to neuropsychology tends to focus on the brain and ignore its relationship with the environment. But in this case, we cannot understand the development of the human mind as it develops during constant interactions between the brain and environment. The results of multiple studies convincingly demonstrate that particular experiences with environment are reflected in brain processing, e.g., a canonical study of an increased hippocampus in London taxi drivers (Maguire et al., 2000); a study by Gaser and Schlaug (2003) on differences in brain structure associated with music training (for a detailed discussion, see Kotik-Friedgut and Ardila, 2014). Importantly, since human environment is a cultural one, an ecologically valid neuropsychology should incorporate disciplines such as anthropology and semiotics, as these disciplines explain the cultural regularities and cultural environment that the human brain reacts during development (Toomela, 2014b). The discipline of cultural neuroscience (Chiao, 2009) specifically focuses on the relationship between brain development and culture. It investigates differences in the brain processes of participants with varied cultural backgrounds (Han and Humphreys, 2016). We believe that the Vygotskian tradition in cultural-historical neuropsychology is in line with recent developments in brain research. For Vygotsky, culture is defined as an environment of sign and language (Vygotsky, 1929/1994; Vygotsky, 1932/1960; Vygotsky, 1935/1994). In cultural development, the sign is “internalized” and becomes a part of cognition. A human mind, according to Vygotsky, starts with a cultural line of development, when children do not simply learn knowledge from adults but internalize human-specific cultural tools – linguistic signs. As Bakhtin and Holquist (1981) notes “becoming a human being … is the process of selectively assimilating the words of others” (p. 341). Based on the conceptualization of cultural tools by Vygotsky (contrary to more traditional socio-cultural schools), it follows that a child can be enculturated from birth. Children are not only born in a human-created environment, but in a linguistically mediated environment that becomes internalized through development. This notion was crucial for Vygotsky. Similarly, an answer to the question of whether animals have culture would differ depending on how one defines culture. This answer can be affirmative if culture is defined as the manufacturing and usage of tools and the ability to modify the environment in which an animal lives (Snowdon, 2017). However, to Vygotsky, the answer would likely be negative, as for him, culture is mainly a semiosphere, socially shared information coded in symbols (Toomela, 1996). In our opinion, Vygotsky’s focus on social processes as sources for individual cognitive development are in line with the modern approaches to cultural research treating culture as inseparable from human biology and recognition of the fact that an individual brain is immersed in a world of social environment. Not surprisingly, contemporary attempts to study culture in a more comprehensive way have led to the emergence of interdisciplinary fields of neuroanthropology (Dias, 2010), neuropsychology (Brickman et al., 2006), neurosociology (TenHouten, 1997), and psychological anthropology (Bock and Leavitt, 2018).

## Conclusion

In this paper, we sought out to demonstrate that Vygotskian thinking can be applied to contemporary psychological research and there is a potential for such an application. To this end, we first reviewed historical, social, and temporal contexts of Vygotskian work. Knowledge of these contexts is useful for understanding aspects of Vygotskian thinking. Following this review, we outlined two core principles transcending every aspect of Vygotskian theory, which in our opinion have the most profound implication for understanding his approach. We suggest that these principles are useful for modern day psychology and are in fact developed by many researchers, although such application does not necessarily rely explicitly on Vygotskian work. Consequently, we discuss how these core principles can be applied to modern psychological research and can assist psychology in solving problems that are still important for the discipline (just as they were important for Vygotsky). In doing so, we intentionally do not provide our readers with direct precise instructions on how Vygotskian approach can be applied to their work. We maintain that our readers are in a better position to develop specific applications of Vygotskian work to their areas of interest. Our goal was to outline potential avenues for such applications and to invite our readers to further dialog on questions that were of interest to Vygotsky himself (such as development and structure of the mind, and the role of culture and language in it).

In conclusion, we suggest that although Vygotsky’s research took place a century ago and every branch of science has made tremendous advancements since then, some of the questions he was working on are still left unanswered. Despite limitations and outdated aspects of his work, there is a potential for incorporating Vygotskian thinking into modern psychological research across disciplines.

We would like to leave the readers with the following reflection. Vygotsky envisioned the success of psychology as a science only if it sustained its holistic and interdisciplinary nature:

Such a system [of an interdisciplinary holistic psychology] has not yet been created. We can say with confidence that it will not arise out of the ruins of empirical psychology or in the laboratories of reflexologists. It will come as a broad biosocial synthesis of the theory of animal behavior and societal man. This new psychology will be a branch of general biology and at the same time the basis of all sociological sciences. It will be the knot that ties the science of nature and the science of man together. It will therefore, indeed, be most intimately connected with philosophy, but with a strictly scientific philosophy which represents the combined theory of scientific knowledge and not with the speculative philosophy that preceded scientific generalizations (Vygotsky, 1925/1997, p. 61; c.f. Yasnitsky, 2011b).

While a comprehensive reconstruction of Vygotsky’s work is yet to be completed, many of the questions he was working on are still relevant to contemporary research. Indeed, there are new perspectives that support early theorizations of Vygotsky (see Newman and Holzman, 2013; Subbotsky, 2014; Alfredo, 2016). We invite scholars working in these interdisciplinary areas to engage with Vygotskian thinking, to explore aspects of his thinking by consulting the work of Vygotskian scholars, and to develop their own nuanced applications of Vygotsky work in their specific research areas.

## Author Contributions

OV and NB conceived the idea for the article and the scope of the review, discussed the results, and contributed to the final manuscript. OV carried out the main literature review. NB verified the analytical conclusions.

### Conflict of Interest Statement

The authors declare that the research was conducted in the absence of any commercial or financial relationships that could be construed as a potential conflict of interest.

## Footnotes

^1^A complete description of the scientific landscape available to Vygotsky during his lifetime is beyond the scope of this paper. There are some excellent publications on the topic (e.g. Van der Veer and Yasnitsky, 2016a,b; Yasnitsky, 2016a).

^2^This situation might rightfully be viewed as restrictive and limiting to scientific inquiry. However, in our opinion, it would be incorrect to suggest that this context erases any value of research conducted during that historical period. Science has never been free from the ideological and political climate of the time. Consequently, scientists were never completely immune from any given political and ideological climate (Aleksandrov and Kirdina, 2012). We believe that rather than perceiving Vygotsky’s work as limited by existing ideology, it may be more accurate to highlight specific aspects that were shaped by this historical period of time.

^3^Some of the works that showcase the productivity of these collaborations include: Vygotsky and Luria (1993), Vygotsky and Leontiev (1932), and Leontiev (1931).
